# “It was like nobody cared about what I said?” Iranian women committed self-immolation: a qualitative study

**DOI:** 10.1186/s12905-021-01221-8

**Published:** 2021-02-19

**Authors:** Nader Aghakhani, Violeta Lopez, Naser Parizad, Rahim Baghaei

**Affiliations:** 1 Patient Safety Research Center, Clinical Research Institute, Nursing and Midwifery Faculty, Urmia University of Medical Sciences, Campus Nazlu, 11 KM Road Seru, 575611-5111 Urmia, West Azerbaijan Iran; 2 School of Nursing, Hubei University of Medicine, Shiyan, China; 3 School of Nursing, University of Tasmania, Hobart, Australia

**Keywords:** Iran, Qualitative, Self-immolation, Survivors, Women

## Abstract

**Background:**

Suicide-attempts have increased across the world and have become higher among females. There has been a high prevalence of self-immolation in Iran, mostly young married women admitted to the burn centers. This study aimed to explore the factors and experiences of self-immolation in Iranian married women to develop prevention strategies to prevent the personal, social, and economic impacts of suicide and suicide attempts.

**Methods:**

A qualitative descriptive approach using open-ended, in-depth, face-to-face interviews was conducted in a purposive sample of 16 married Iranian women aged 16–40 years in the burn centers in Urmia city, a place in northwest Iran. Conventional content analysis was used to analyze the data.

**Results:**

Three themes emerged from the data, including (1) antecedents of self-immolation, (2) suicidal ideation method, and (3) pathway to recovery. Each of these themes is supported by sub-themes.

**Conclusions:**

The study highlights the demand for health professionals to support self-immolation survivors to continue their normal lives. According to survivors’ needs, a comprehensive supportive program is recommended to support their pathways to recovery in all its complexities. Health professionals should also not forget that the survivors’ family also will need help to overcome this trauma. A family counseling program may also be provided.

## Background

Recently, suicide as the cause of mortality has increased across the world [1]. The countries of the former USSR have the highest suicide rates [2]. Pakistan has the highest suicide rate, while Bahrain has the lowest suicide rate among the countries in the region [1]. In Iran, the suicide rate of 6.2 per 100,000 was reported in 2003 but has increased to 9.9 per 100,000 in 2017 [3]. However, different suicide rates have been reported to vary from 0.72 per 100,000 in Qom province to 271.1 per 100,000 in West Azerbaijan province in Iran [4]. Suicide can be viewed as an interaction of mental, physiological, clinical, social, clinical, cultural, and protective factors. [5]. Suicide is a psychosocial response to a hostile environment, feelings of despair, addiction such as alcoholism, post-traumatic stress disorder, major depression, schizophrenia, agitation, and social isolation. Its causing factor is often correlated with financial difficulties or interpersonal problems [6]. Family conflicts may result in suicidal ideation in young people in different ways, such as ineffective coping strategies, lack of hope in life [7], lack of positive personality capital in the form of hope, self-efficacy, resilience, and optimism about the future [8].

According to the community's cultural context, there are many methods of suicide, including hanging, poisoning, suffocation, and self-immolation [9]. Self-immolation, or self-burning, is defined as a deliberate and willing sacrifice of oneself, often by fire, which is the common violent, tragic, and dramatic type of suicide in middle-aged adults [10, 11]. It has also been observed in young married women in other Asian settings, including Iran, Iraq (Kurdish Regions), Afghanistan, Pakistan, India, and Sri Lanka (Eastern Province) [11–13]. In Iran, 25–41% of all suicides are from self-immolation, making Iran one of the countries with the greatest burden of mortality and morbidity in the world [12].

The prevalence of self-immolation in Iran makes it a socially problematic health challenge. The rate of suicide in women is higher than in men, and, unfortunately, the number of suicide attempts among adolescents in Iran has also recently increased [13]. Most studies regarding female self-immolation are quantitative studies covering demographic characteristics; however, this social event cannot be generalized to other cultural and social settings in Iran and must be fully investigated. The victims’ motives, sociocultural issues, and experiences need to be elucidated. This study was conducted only among Iranian married women to determine the factors leading to their suicide attempts. Self-immolation is a public and preventable health problem that may have resulted from cultural, psychological, religious, emotional, social, and economic complications. The consequences of suicide may have long-lasting effects on the families and friends of suicide survivors [14]. Prevention of suicide attempts demands collaboration among health-care providers, patients, and their family members to promote the development of new solutions that can help save lives [15].

## Methods

### Design

A qualitative descriptive study using in-depth interviews was employed in this study. Purposive sampling was used to recruit participants. A total of 16 participants consented to participate in a face-to-face and in-depth interview. Participants were recruited from the West Azerbaijani province in Iran. The inclusion criteria were married female survivors of self-immolation. The women provided consent as well as their husbands and/or family members. This is important as the male researcher conducted the interview. Participants whose families/spouses did not permit the interview were excluded. The interviews were conducted from April to August 2019 and lasted 45–55 min using the interview guidelines. The interview guideline was based on the integrated motivational–volitional (IMV) model of suicidal behavior [16]. The IMV model is a three-phase biopsychosocial framework consisting of: (1) pre-motivation phase—background factors and triggering events, (2) the motivational phase: the emergence of suicidal ideation, and (3) the volitional phase: from suicidal ideation to suicide attempts/suicide. Understanding the social and environmental context of suicide risk is important [17]. The model also focuses on the psychological processes that lead to suicidal ideation, beginning with feelings of defeat, rejection, loss, and having no social support [18], as well as access to the means of suicide and exposure to the suicidal behavior of family or friends [19]. We aimed to explore the reasons why participants attempted self-immolation, the consequences, and how they felt about the aftermath. The open-ended questions asked were: “Please tell me the reasons why you attempted suicide.” “What method of self-immolation did you use, and why?” What are your feelings now after the incident?” Based on the participants’ answers, probing questions were asked to gain a deeper understanding of the subjects. These questions include: What do you mean? Would you please, tell me more about this? The research team discussed the interview questions and was piloted to two women who had experienced self-immolation. Modifications were made only for clarity. The lead researcher conducted all interviews and wrote memos after each interview to capture any nuances observed as well as to set aside his own feelings and assumptions about the focus of the study and maintain an open-mind instead of being judgmental. All interviews were conducted in Persian, audio-recorded, and conducted in a quiet private place and at a time convenient for the participants. Data collection ended when data saturation was met.

#### Data analysis

Data analysis was conducted using the six steps conventional content analysis approach [20], including (1) being familiar with transcribed data by immersion and detecting primary code by reviewing (2) creating primary codes through reading line by line, (3) searching for and identifying themes, (4) reviewing themes to find the relationship between themes and sub-themes, (5) labeling themes and sub-themes, (6) formulating the final report of the analysis. The lead researcher analyzed data using MAXQD10 software. Data were transcribed verbatim and were checked by two researchers for accuracy. The initial codes were obtained, noting their differences and similarities. These codes were then organized into larger categories.

#### Rigor

Lincoln and Guba’s criteria were used to ensure the trustworthiness of the study [21]. The credibility was ensured by prolonged engagement with data, participants’ feedback, and expert review. To achieve conformability, a detailed report of the research process was provided. The researcher reflected and reported any predispositions that might have affected the objectivity of the study. This process and the interview transcriptions ensured that the researcher maintained an awareness of potential biases during the data analysis phase. We also included a comprehensive description of the study procedures to strengthen the results' dependability and allow repetition of the study by other researchers. The research process was repeated step by step, described in detail, and other research team members confirmed them to assure dependability and transferability. To avoid unconscious bias, we asked two female colleagues who were experts in qualitative methods to review the transcripts and coding structure. They appraised the analyzing process, and we reviewed their comments and recommendations and addressed the possible issues.

## Results

### Participants’ demographic profile

The participants were 16 young married women survivors of self-immolation in West Azerbaijan province with a mean age of 26.73 (SD = 2.58; range 16–40) years. Half of them completed primary education, half were housewives, seven were employed, and only one was a student. Most of them had arranged marriages and resided in rural areas.

### Qualitative findings

Three categories emerged from the data, including (1) antecedents of self-immolation, (2) suicidal ideation method, and (3) pathway to recovery, as shown in Table 1. These four themes are supported by sub-themes, and participants’ quotes are denoted as, for example, P1 (Participant number 1) to protect their identity.

**Table 1:** Categories and supporting sub-categories that emerged from the data

Categories	Sub-categories
Antecedents of self-immolation	Overwhelming family situation and rules Feelings of disappointment, anger deprivation, depression, shame, and grief A way for family to feel guilty
Suicidal ideation method	A final solution to their sufferings Uncertainty about the method of suicide Feeling trapped
Pathway to recovery	Blaming themselves for their mistakes An awakening process Seeking family support to start a new life

## Categories 1: antecedents of self-immolation

Many factors led to women deciding to self-immolate. These factors were mostly due to psychological factors experienced being in an unbearable family situation, and feelings of helplessness that nobody cared to listen to them. The following three sub-categories further described these antecedents.

### Overwhelming family situations and rules

Being controlled by the obligatory rules of the family was reported as overwhelming. Such feelings induced psychological problems. The women experienced tensions, negative moods, conflicts, and the inability to relate to others. In some instances, they also experienced domestic violence from their husbands. Suicide was their choice to free themselves from their intolerable situation.

There are many days when my husband yelled at me and told me that I am so stupid and ugly. He told me he did not want to marry me. I tried to change his attitude, but I found him reluctant to speak with me. I was depressed and hid in another room to be safe from his verbal blame and physical violence. I think that suicide is the only way that I could escape from him. (P1)

The women not only experienced unbearable situations from their husbands but also with their own families. They reported that the traditional Iranian patriarchal family dominates decisions about their future, even arranging whom they have to marry. The women reported being deprived of making their own decisions, further exacerbating their psychological distress.

Traditionally, in our family, my father makes decisions for everything, even our life or marriage. The independence of the daughter and her fate and expectations are ignored by male family members and elders, which often creates an unpleasant situation. Nobody dares to argue about my future. Nobody cared what I have to say. (P12)

### Feelings of disappointment, anger, deprivation, depression, shame, and grief

Participants expressed feelings of disappointment, anger, deprivation, depression, shame, and grief due to cultural and social contexts. In their minds, life was considered a terrible circumstance without any hope, and they felt helpless. They thought this situation put an end to their freedom and believed that this kind of life equals death.

Even thinking of my life is frightening; imagine someone telling you that you have to marry a man that you do not love and continue your life with him! I was constantly crying for my misfortune, deprivation and loneliness. My father told me I do not have the right to return to our home if I get a divorce, and my family will be ashamed. It was terrible, and I was disappointed and ready to die. (P.9)

The participants also felt deprived and became more depressed and jealous of other women who have more freedom and have a better life than them.

When I compare my life with other women in my family, I feel hatred towards myself, my husband, and my circumstances. I do not have anything. We are too poor and have no bright future. (P.3)

### A way for family to feel guilty

Other participants resorted to self-immolation to make their family feel guilty for not letting them pursue their dreams. They reported that if their families feel guilty, they can have the freedom to pursue their dreams.

I was a very clever student, but my family did not let me go to high school. Quarreling with my family made me think about suicide every day, and I wanted to make them feel guilty. (P.10)

## Category 2: ideation of self-immolation method

Participants reported no hope in overcoming their unbearable sufferings, so decisions were made without thinking of the consequences. They know some of their family members who resorted to self-immolation, so why not also follow what they did. They had the impression that it is okay, after all. The following three sub-categories further described the participants’ ideation.

### A final solution to their sufferings

Choosing self-immolation was a final solution to stop women’s suffering. It was difficult for them to think that they can continue their life in difficult circumstances. This state was worse for the women who were married at an early age and those whom their family and relatives did not support.

Nobody cared what I said. So, I decided to burn myself because I thought this method would kill me faster than the other methods. (P.10)

My last hope was my parents. When I realized that they were no longer supporting me, I gave up. (P.4)

### Uncertainty about the method of suicide

Some participants stated that despite the decision to commit self-immolation, they had little information about the means of self-immolation and had no idea how to attempt self-immolation. They also were unsure what method is the best choice for them to kill them fast and suffer less. Some participants shared their experience as follows:

I decided to burn myself but did not know how!? I suddenly poured gasoline on my head and then set my own body on fire. (P.14)

### Feeling trapped

Some participants talked about how they felt trapped at home and not allowed to go out to prepare the means of suicide. Thus, they had no choice but to burn themselves. The availability and easy access to flammable liquids like kerosene and matches made them quickly carry out their intention.

One of the participants voiced:

… I could not go out to get pills. So, self-immolation for me was the only way to kill myself. (P3)

## Category 3: pathway to recovery

Experiences participants shared made them think about how they could recover even though they knew it would be a long process. They accepted that they have to be patient as they have learned a lot from this painful experience. The following three sub-categories further described the aftermath of their suicide attempt.

### Blaming themselves for their mistakes

Participants mentioned that they accepted their own mistake after suicide. All of them voiced that they felt regret and would not repeat this wrong act. They believed that suicide resulted from being impatient; therefore, they blamed themselves for committing it.

I want to marry again. Now I am very regretful for not revealing my secrets. What a stupid thing I have done! I will never do that again. (P.6)

I tried to punish my family, but unfortunately, I punished myself. I made the worst mistake of my life. (P.11)

Some of them considered their wrong behavior as the reason for their problems. They blamed the culture and society, but they also believed that they could have done something to resolve their issues.

I had no problem in my marital life. One day, I argued with my husband; I was so nervous, so I decided to kill myself. At present, I am ashamed and asked myself why I did so? (P.2)

### An awakening process

The aftermath of self-immolation had been an awakening process for the participants. They reported that they should have done more reflections about the root cause of their problems. Being open about the problems they were experiencing may have abated their self-immolation.

Thinking of my life was frightening. Imagine your family telling you all the time what to do and not do even if you are not happy and opposed to it. Now, after my self-immolation, I tell myself to be brave and open up what I feel and what I want to do. Hopefully, they will listen. (P.9)

### Seeking family support to start a new missing life

Participants were worried about their uncertain future after surviving the self-immolation. Fear of being alone, failing to continue their normal life, and low self-esteem were the main reasons for seeking support. The participants reported that having family support can help them to return to a normal life. Family acceptance was very important for the participants after self-immolation, as they have learned from their mistakes and understood why they did it.

Now I did that [burn myself. I should never give up. My father accepted his mistakes and promised to help me get back on my own feet. (P.7)

After this mistake happened, I became more dependent on my family… I felt that they are the only ones that can help me… Now I feel they have forgiven me for my foolish deed. (P.8)

## Discussion

This study explored the experience of self-immolation in Iranian married women. Based on our participants’ experiences, we explored some antecedents or pre-motivational factors that pushed our participants toward deliberate self-immolation. They talked about their overwhelming family situation and strict rules that gradually took them toward self-immolation. The conflict between family members, especially husbands and wives, was one of the pre-motivating factors our participants voiced when they performed self-immolation to resolve their marital conflicts. Another Iranian research also confirmed that marital conflict is a core cause of self-immolation [22]. In previous studies, family conflicts were also a factor to self-immolate because of traditional cultural customs and values. [7, 23]. Another study found that married women were at the greatest risk of suicide by burning, mostly due to quarrels with a family member, a relative, or a friend [24].

Based on the present interviews, it was observed that ignoring women’s rights by their family and society, along with a dominant cultural and social context’s restriction and prohibitions, triggered self-immolation [25]. They had no choice about what they like to do or even whom they want to marry, which deteriorated even more after they got pregnant. A study found that forced marriage led to suicide even without psychological problems [26]. Suicide attempts resulting from forced and early marriage were also reported in two separate studies in Turkey and Sri Lanka [27, 28]. Domestic violence and marital conflicts in rural areas with unfair male dominance consisted of unresolvable problems for our participants. In line with previous studies, our participants who resorted to self-immolation were less educated, and half of them just completed primary education. Low literacy is among other negative psychosocial factors such as high family conflict and lack of social support that may put the young married women at higher risk of self-immolation [28]. Their overwhelming family situation and strict rules made them feel disappointed, angry, deprived, depressed, shame, and grief. These feelings were triggered when the cultural and social context of obedience to the family’s male members was expected [27]. When participants could not solve their problems, they tried to find a way to make their family feel guilty. This motivated these suffering women to burn themselves to punish their families. These factors and triggering events support the pre-motivation phase in the IMV model [16].

Participants entered the motivational phase after they had a hard time handling the stressful situation. As reported by our participants, a self-immolation attempt is a final solution to their sufferings. Most of our participants believed that they performed self-immolation to resolve marital conflicts, forced marriage, and the age gap between them. They deliberately burn themselves to overcome their distressing situation, mainly enforced by family-arranged marriage. For some women, the option of self-immolation was a way not only to get out of their prison-like situation but also to make their husbands and family feel guilty about the way they were treated [29]. Some participants were hesitant about choosing the means of suicide because they did not have enough information about suicide methods. Since the self-immolation method is so painful and affects the family further, some women use it to induce more guilt in their family; namely, they punish their husbands or families and take revenge for their behavior [30]. Some women probably take this suicide method because it is an easy method and they do not need to leave the house to provide some materials to commit suicide. Our participants felt trapped and deprived of all freedom as some were not allowed to leave the house. The easy access and availability of some flammable liquids and matches made the suffering women burn themselves*.* Thus, our participants chose self-immolation because it was the fast and easiest way and sometimes the only way to kill themselves with less suffering [24, 30]. According to the IMV model, our participants' psychological issues lead to suicidal ideation, beginning with feelings of defeat and entrapment [18].

The final act of self-immolation supports the phase three volitional phase of the IMV model [16]. Our participants had access to the means of suicide and were exposed to the suicidal behavior of their family members, who also resorted to self-immolation [19]. However, what was lacking in the IMV model was the aftermath of the attempted self-immolation as our participants had no real intention to die and did not realize that self-immolation is a dangerous means of suicide with many adverse consequences [31]. Our participants accepted that they regretted performing self-immolation and learned the best way to recover. However, there are also examples in which people in challenging situations can choose to stay alive or perform suicide. A study reported that people who feel that they have a certain purpose in life, e.g., a child to care for, choose to stay alive even if their circumstances are at odds [32]. They tried to seek family support and satisfy them by cleaning their dark past and taking proper actions to make up for their mistakes. These people try to establish a warm and supportive relationship with their family members to be more resistant to stress and more easily adapt to existing conditions [23, 33]. Other studies have confirmed the advantages of social support in self-immolation survivors through support from family members and friends [32, 34]. Thus, it is important that women who survived self-immolation be supported by their family and other support networks.

## Limitations

The study was limited because of the small sample size and the need for husbands and the family to consent for the young married women to participate in the study. This could have hampered the in-depth accounts of their experiences. However, even with this small sample size, the findings have provided insightful data on the reasons for, consequences of, and recovery from attempted self-immolation.

## Conclusions

The study revealed that married women who have been under psychological pressure from their families and whose needs were ignored attempted suicide by self-immolation. They tried to make their voices heard by the family and society. If their voices were ignored, they would suffer from anger, despair, hopelessness, and depression. These women’s last resort to get rid of stress and suffering was to set foot on the road to self-immolation. The study highlights the need for health professionals to focus on self-immolation survivors to encourage them to continue normal lives. According to survivors’ needs, a comprehensive supportive program is recommended to support their pathways to recovery in all its complexities. Family members in a male-dominated society, husbands, fathers, and brothers are urged to take part in protective practices towards self-immolation and other suicide methods. Health professionals should also not forget that the family of the suicide survivors will also need help to overcome this trauma. In addition, preventive strategies such as family counseling services and teaching coping skills to women in stressful family situations may reduce the risk of suicide by self-immolation.

## Data Availability

The datasets used and/or analyzed during the current study are available from the corresponding author on reasonable request.
